# The effect of mobilization with movement on pain and function in patients with knee osteoarthritis: a randomized double-blind controlled trial

**DOI:** 10.1186/s12891-019-2841-4

**Published:** 2019-10-18

**Authors:** Hani A. Alkhawajah, Ali M. Alshami

**Affiliations:** 1 Department of Physiotherapy, King Fahd Hospital of the University, Imam Abdulrahman Bin Faisal University, P.O Box 40244, Khobar, 31952 Saudi Arabia; 2 Department of Physical Therapy, College of Applied Medical Sciences, Imam Abdulrahman Bin Faisal University, P.O. Box 2435, Dammam, 31441 Saudi Arabia

**Keywords:** Hypoalgesia, Manual therapy, Pressure pain threshold, Quantitative sensory testing

## Abstract

**Background:**

Few studies have investigated the effects of mobilization with movement (MWM) in patients with knee osteoarthritis (OA) compared to other procedures. Sham procedures are generally more appropriate control than using no or usual treatments. Moreover, studies investigating the widespread hypoalgesic effects of MWM in patients with knee OA are lacking. The aim was to investigate the effect of MWM on function and pain in patients with knee OA compared to sham MWM.

**Methods:**

This is a randomized double-blind (patients and assessor) controlled trial. Forty adult patients with knee OA of grade II and above were recruited to receive either MWM treatment or sham MWM for the knee. The outcome measures included the following: a visual analogue scale (VAS) for pain, the pressure pain threshold (PPT) test, the Western Ontario and McMaster Universities Osteoarthritis (WOMAC) Index, the timed up and go (TUG) test, knee strength and knee range of motion (ROM). The measurements were taken at baseline, immediately after intervention and 2 days later.

**Results:**

Compared with sham MWM, MWM resulted in greater immediate improvement in pain [mean difference (95% CI): − 2.2 (− 2.8, − 1.6)], PPT at both the knee [176 (97, 254)] and shoulder [212 (136, 288)], TUG time [− 1.6 (− 2.1, − 1.1)], knee flexor strength [2.0 (1.3, 2.7)] and extensor strength [5.7 (4.1, 7.2)] and knee flexion ROM [12.8 (9.6, 15.9)] (all, *p* < 0.001) but not knee extension ROM [− 0.8 (− 1.6, 0.1)] (*p* = 0.067). After 2 days of intervention, patients who received MWM also demonstrated a greater improvement in pain [− 1.0 (− 1.8, − 0.1)], PPT at the shoulder [107 (40, 175)], TUG time [− 0.9 (− 1.4, − 0.4)], knee flexor strength [0.9 (0.2, 1.7)] and extensor strength [2.9 (2.1, 3.9)] and knee flexion ROM [8.3 (4.7, 11.9)] (all, *p* ≤ 0.026). However, WOMAC scores and knee extension ROM showed no evidence of change at any stage after intervention (*p* ≥ 0.067).

**Conclusions:**

MWM provided superior benefits over sham MWM in terms of local and widespread pain, physical function (walking), knee flexion and extension muscle strength and knee flexion ROM for at least 2 days in patients with knee OA.

**Trial registration:**

ClinicalTrials.gov (NCT02865252), registered on August 12, 2016.

## Background

Osteoarthritis (OA) is the most prevalent form of joint arthritis [1]. Knee OA accounts for pain and functional disability in 19.2–27.8% of people aged > 45 [2, 3]. Approximately 37% of people aged ≥60 had knee OA on radiograph [4]. Data on the prevalence of OA in Arabic countries is scarce [5]. However, in Saudi Arabia, a cross-sectional study found that of 300 patients, 53.3% of men and 60.9% of women demonstrated radiographic features of knee OA. Eighty per cent of these patients reported knee pain [6].

There is no known cure for OA [7]. The management of knee OA aims to control pain while improving function and quality of life [8]. The most common medical interventions include pharmacological agents and joint replacement surgery. However, the latter is high risk, especially in older patients [9, 10]. In contrast, other less invasive treatments, such as targeted manual therapy and exercise, are cost-effective and can be safely administered to older patients with OA [7]. Although clinical guidelines report that the efficacy of manual therapy and electrotherapeutic modalities is unclear in patients with knee OA [11], recent high-quality studies [12–14] have found that manual therapy decreases pain, increases range of motion (ROM) and improves physical function.

Mobilization with movement (MWM), which is a type of manual therapy with hypoalgesic effects, increases joint ROM, enhances muscle function and treats specific pathologies [15]. MWM is effective in the management of patients with tennis elbow [16, 17], ankle sprains [18, 19], shoulder impingement [20] and hip OA [21]. Other types of manual therapy, namely antero-posterior glide of the tibia on the femur, produce both local and widespread hypoalgesic effects in patients with knee OA [22].

To our knowledge, three studies have attempted to investigate the effects of MWM in patients with knee OA. These studies were either case series [23] or randomized controlled trials (RCTs) [24, 25] that used other treatment procedures in addition to MWM. Sham procedures more clearly distinguish the efficacy of a new procedure beyond the placebo response [26]. In addition, studies that particularly investigate the widespread hypoalgesic effects of MWM in patients with knee OA are lacking. Therefore, the aim of this study is to investigate the immediate and short-term effects of MWM on function and local and distant pain in patients with knee OA compared to sham MWM. This study will serve as the basis for long-term RCTs in the future. The current study is part of a larger study of a master’s thesis that has two phases. Phase one is presented in the current study, and phase two aims at evaluating the effect of MWM in a group of patients who demonstrate features of central sensitization.

## Materials and methods

### Study design and setting

This double-blind randomized controlled trial was conducted in the Department of Physiotherapy at King Fahd Hospital of the University (KFHU). The study was retrospectively registered with ClinicalTrials.gov (NCT02865252) and approved by the Institutional Review Board (IRB) (IRB-2014-04-323) at Imam Abdulrahman Bin Faisal University. The participants provided their written informed consent to undergo the treatment and to have their data used in the study. The study was carried out with CONSORT reporting guidelines [27] in mind.

### Sample size determination and participants

Sample size calculation was performed using statistical software (G*Power 3.1) with the following combination: analysis of variance, repeated measures, within-between interaction, medium effect size (f) of 0.25, alpha level of 0.05, power (1-β) of 80%, correlation (*r*) of 0.5, with 2 groups and 3 measurements (time points) and non-sphericity correction (Є) of 1. The estimated desired sample size was 28. A minimum of 18 patients per group was needed taking into consideration a 20% attrition rate.

Patients with knee OA who attended KFHU were recruited. Patients were diagnosed at the orthopaedic clinic and referred to the Department of Physiotherapy. Patients who were willing to participate in the study were screened for eligibility. The patients were included in the study if they were men or women aged ≥40, had unilateral or bilateral knee OA with a Kellgren and Lawrence (K&L) grade ≥ 2 [28], fulfilled the classification criteria of the American College of Rheumatology for knee OA [29], reported peak knee pain of > 3 on a visual analogue scale (VAS) over the previous 24 h and were able to walk ≥6 m. Patients were excluded if they had knee or lower limb surgery, had received an intra-articular corticosteroid or hyaluronic acid injection within the past 6 months, reported current or past (within 4 weeks) oral corticosteroid use, had inflammatory or neurological disorders, had altered sensation (to cold, heat, or pressure) around their knee, exhibited cognitive difficulties, had low back-related leg pain or had any contraindication to manual therapy.

Blinded to the allocation, participants were recruited consecutively and randomly allocated to either a treatment group (MWM) or a sham group (sham MWM) using a simple randomization procedure. A receptionist who had no other involvement in the study generated the sequential numbers using an online randomization website (https://www.graphpad.com/quickcalcs/randomize1.cfm). Forty numbers were uniquely randomized in equal number to two different groups, and each number and its allocated group was written on a piece of paper and concealed in an opaque envelope. The receptionist informed the treating therapist (principal researcher) about patients’ allocation after the baseline measurements were taken. Patients were asked to attend on two occasions. The first visit took approximately 2 h, during which measurements were taken at baseline, the intervention was delivered and immediate post-intervention measurements were taken. The second visit occurred 2 days later and lasted 30–45 min for measurement only (short-term effect). The testing procedures were identical for each patient, except that patients in the sham group received sham MWM.

### Intervention

A physiotherapist (principal researcher), blind to the measurements until data analysis, who has 10 years of clinical experience and who is a certified Mulligan practitioner trained in the use of MWM administered treatments to all patients. MWM techniques were performed using a sustained medial, lateral, anterior, posterior or rotation glide of the tibia during active knee flexion and extension. The details of these techniques have been described previously [30]. The glides were tested in all possible directions while the patient was in the supine position using the following order: frontal plane (medial/lateral), sagittal plane (anterior/posterior) and then rotation. The glide direction that relieved pain to the lowest level and improved knee range most was selected as the glide for treatment. If the movement was not painful, overpressure was added at the end range. The glide direction was examined in weight-bearing if there was no pain in the supine position. If several glide directions showed similar effects in the supine position, these tests were performed in a weight-bearing position to determine the most effective glide direction [23].

In the treatment group, the therapist applied the glide force on the tibia with the knee in mid-range. Then this force was maintained while the patient was flexing and extending the knee to full range. Overpressure was performed at the end range. The MWM treatment technique was repeated 10 times for three sets [23].

In the sham group, the patients were handled similarly to those in the treatment group, but they did not take the glide of direction. Alternatively, the therapist’s hands were lightly touching the knee skin without pressure, one hand on the tibia and one on the femur. Active knee flexion and extension movements, however, were performed 10 times for three sets.

### Outcome measures

An independent experienced physiotherapist (assessor) from the Department of Physiotherapy (with > 5 years of clinical experience) who was blinded to the allocation of the patients collected the demographic data and baseline measurements of all outcome measurements. Then, the assessor left the room to remain blind to conditions while the principal researcher applied either MWM treatment or sham MWM interventions according to the patient’s allocated group. After that, the principal researcher left the treatment area and the assessor performed the outcome measurements immediately after the intervention in a similar manner to the baseline measurements. Patients were asked not to discuss their treatment experience with the assessor. Two days later, the assessor performed the outcome measurements again to assess short-term effects [31].

### Primary outcomes

#### Visual analogue scale (VAS)

Current pain intensity was measured using a 10-cm VAS with end points marked ‘no pain’ and ‘worst pain imaginable’. The VAS is a valid and reliable measure of pain intensity [32–35].

#### Pressure pain threshold (PPT)

A digital pressure algometer (Somedic AB, Farsta, Sweden) was used to quantify pain intensity in accordance with similar clinical studies [16, 22]. This measure has demonstrated high reliability with an intraclass correlation coefficient [ICC (2,3)] of 0.97 [36]. PPT is the lowest stimulus intensity at which a person feels mechanical pain. Increased values of PPT may indicate hypoalgesia or decreased response to mechanical pain stimuli [37].

The most tender point on the medial aspect of the participant’s affected knee was palpated, marked and photographed to ensure standardization between measurements. With the participant in a side-lying position, a 1 cm^2^ algometer probe was used to apply pressure at 90^o^ of knee flexion perpendicular to the skin at a rate of 40 kPa/s. Participants were asked to press a button when the sensation of non-painful pressure turned to become painful. The PPT value was recorded at this point. PPT was also examined on the middle deltoid, 10 cm away from the acromion of the ipsilateral shoulder, to investigate any widespread changes in sensitivity at a distant site. Three measurements were performed in each area (knee and shoulder), and the mean value was recorded for analysis. A rest period of 20 s was given after each measurement.

#### Western Ontario and McMaster universities osteoarthritis (WOMAC) index

This self-administered questionnaire was presented using a five-point ordinal scale with five categorical responses (numerical value of 0–4). WOMAC was designed to measure perceived pain, stiffness and dysfunction. High WOMAC scores reflect greater severity across the three measured domains [38]. WOMAC has moderate-to-excellent reliability and validity to test pain, stiffness and function, especially in patients with hip or knee OA [38–40].

#### Timed up and go (TUG)

This common test used to assess walking ability has been described in detail previously [41]. TUG has showed high inter- and intra-rater reliability [ICC (2,1) = 0.96–0.97] in an arthritic population [42]. One practice trial was performed prior to testing. Three measurements were performed, and the mean value was recorded for analysis. A rest period of 15 s was given after each measurement.

### Secondary outcomes

#### Hand-held dynamometer

A digital dynamometer (Commander Power Track II, JTECH Medical Industries, Midvale, USA) was used to examine muscle strength and force development. It has good-to-excellent intra- and inter-rater reliability [ICC (2,1) ≥ 0.70] and moderate-to-excellent validity to test muscle strength [43, 44]. The strength of knee flexors and extensors in kilograms was measured while sitting with the knee at 90^o^ flexion. To provide resistance throughout the range, the hand-held dynamometer was placed on the distal tibia anteriorly when examining knee extensors and placed on the posterior ankle when examining knee flexors. Three repetitions were performed in each direction, and the mean value was used for analysis. A rest period of 15 s was given after each repetition.

#### Standard goniometer

A standard goniometer (EZ Read Jammar, Sammons Preston, Warrenville, USA) was used to measure active knee flexion and extension ROM in the supine position. The test was performed three times for each direction, and the mean value was used for analysis. Goniometer measurement demonstrated moderate to high inter-rater reliability (ICCs = 0.59–0.90) [45].

### Statistical analysis

Data were analysed using IBM SPSS for Windows (version 24.0). Descriptive analysis included means, standard deviations, medians and interquartile ranges. Q-Q plot and Shapiro-Wilk test of standardized residuals were performed for checking the normality of residuals. All continuous variables were approximately normally distributed, except for knee extension ROM. For this variable, the assumption was not met even after transformation, but the model residuals were acceptable. Homoscedasticity was tested for WOMAC by plotting a scatterplot of the standardized residuals against the predicted values. Linearity assumption was assessed by plotting a scatterplot of outcome values at follow-ups against baseline values in each treatment group. The scatterplots did not indicate major departure from these assumptions. The primary analysis was performed on an intention-to-treat basis, and all randomised participants were included. For continuous outcomes, the least square means (LS means) and their 95% confidence intervals (CIs) were estimated using a linear mixed model (LMM) for repeated measures with participant as a random effect, baseline score as a covariate [46, 47] and outcomes at two follow-up visits as a dependent variable. This model contained the treatment group, time, baseline-by-time interaction and group-by-time interaction as fixed-effects with an unstructured covariance matrix among time points. For the WOMAC, which was measured with a single follow-up time (2 days), analysis of covariance (ANCOVA) with baseline value as a covariate was used. Mean changes for each group at each time point and mean between-group differences were estimated using appropriate contrasts in the models. All data were regarded as significant at *p* < 0.05 (two-sided).

## Results

**Fig. 1:**
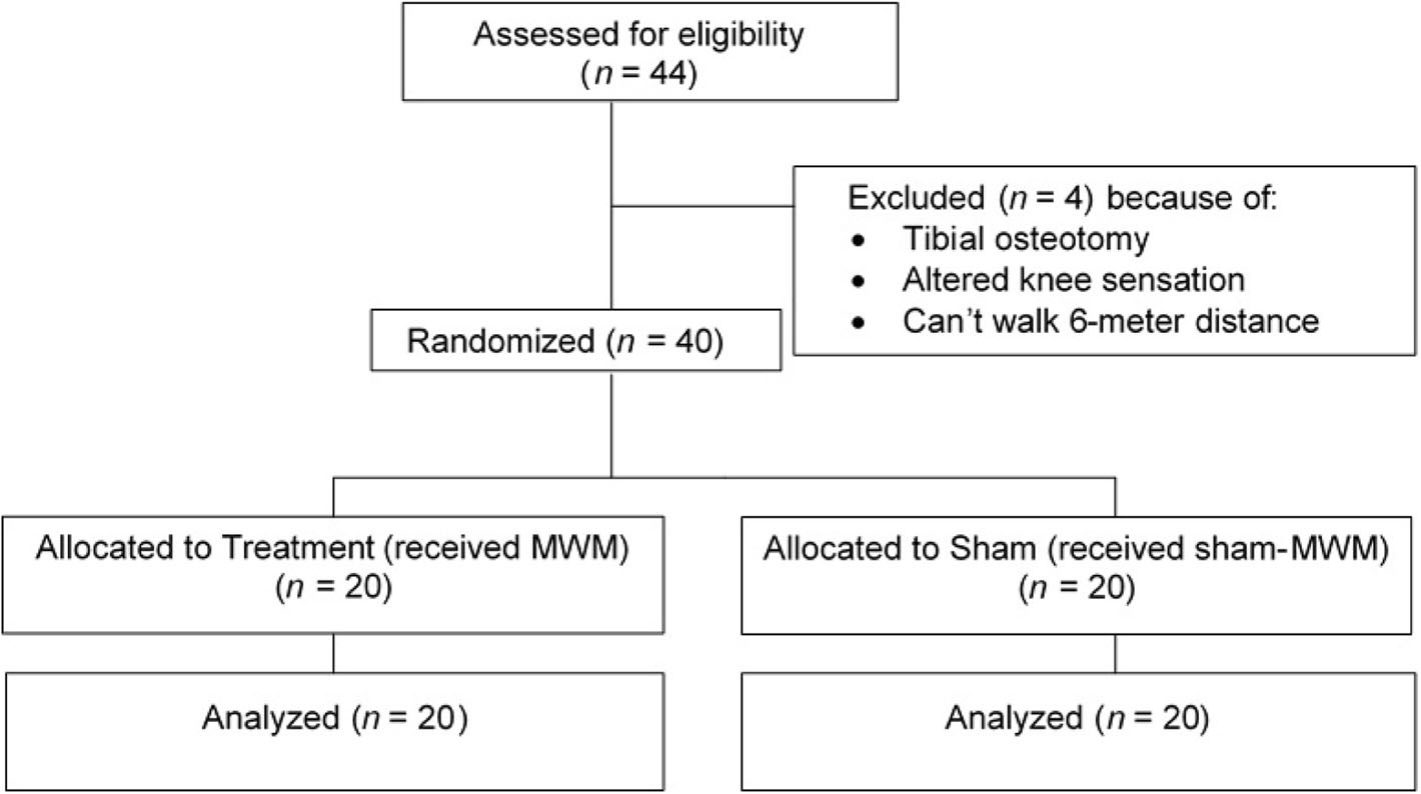
Consort diagram of patients enrolment and randomization

Forty-four patients were screened for eligibility. Forty patients satisfied the criteria. Of the 40 patients, four were excluded because of tibial osteotomy, two because of altered sensation around their knees and one because they were unable to walk a 6-m distance with or without an aid. Figure 1 shows the enrolment and randomization process. Table 1 shows the demographic data of the patients. Table 2 demonstrates the direction of glide applied for the MWM intervention. The medial glide of the tibia over the femur was the most common technique.

**Table 1:** Characteristics of patients in both groups at baseline

	MWM (*n* = 20) Mean ± SD	Sham (*n* = 20) Mean ± SD
Age (years)	56.5 ± 7.6	56.6 ± 8.5
BMI (kg/m^2^)	32.6 ± 7.8	33.3 ± 6.1
Duration of symptoms (months)^a^	51 (46)	48 (42)
VAS (10 cm)	6.5 ± 1.9	5.7 ± 2.0
Gender (male/ female)	13 / 7	12 / 8
Affected knee side (right/left)	6 / 14	10 / 10
K&L knee OA grade (2 / 3 / 4)	14 / 4 / 2	13 / 3 / 4

*MWM* Mobilization with movement, *SD* Standard deviation, *BMI* Body mass index, *VAS* Visual analogue scale, *K&L* Kellgren and Lawrence (1957) grading system, *OA* osteoarthritis

^a^Values are expressed as median (interquartile range)

**Table 2:** Direction of glide chosen for the MWM intervention

Direction of glide	MWM (*n* = 20)
Medial glide	7
Medial + internal rotation glide	2
Lateral glide	3
Lateral + external rotation glide	1
Internal rotation glide	5
Anterior glide	2

*MWM* Mobilization with movement

The group-by-time interaction for the LMM was statistically significant for VAS (*F* = 27.69, *p* < 0.001), PPT at the knee (*F* = 10.86, *p* < 0.001), PPT at the shoulder (*F* = 16.06, *p* < 0.001), TUG (*F* = 21.31, *p* < 0.001), knee flexor strength (*F* = 15.37, *p* < 0.001), knee extensor strength (*F* = 32.46, *p* < 0.001) and knee flexion ROM (*F* = 36.83, *p* < 0.001). This interaction was not statistically significant for knee extension ROM (*F* = 2.30, *p* = 0.115). The results show significantly greater mean changes from baseline for knee flexion ROM in the treatment group compared to the sham group at follow-up visits 1 and 2 [the mean between-group difference was 12.8 (*p* < 0.001) and 8.3 (*p* < 0.001), respectively]. Tables 3 and 4 show the LS mean changes and their 95% CIs in the outcomes of the treatment and sham groups over time estimated using LMM and ANCOVA analyses. The tables also report the difference in the LS mean change between the groups at follow-up visits 1 and 2. Compared to those receiving sham MWM, the patients who received MWM demonstrated an immediate greater decrease in pain, a greater increase in PPT at both the knee and shoulder, a greater decrease in TUG time, a greater increase in knee flexor and extensor strength and a greater increase in knee flexion ROM (all, *p* < 0.001) but not in extension ROM (*p* = 0.067). Two days after intervention, patients who received MWM demonstrated a greater decrease in pain, a greater increase in PPT at the shoulder, a greater decrease in TUG time, a greater increase in knee flexor and extensor strength and a greater increase in knee flexion ROM compared to those who received sham MWM (all, *p* ≤ 0.026). However, no significant differences were found between the treatment and sham groups in PPT at the knee (*p* = 0.142) or knee extension ROM (*p* = 0.499) (Table 3). The ANCOVA revealed no significant differences between the two groups in the total score or any sub-scale of WOMAC (*p* ≥ 0.392) (Table 4).

**Table 3:** Comparison of pain, pressure pain threshold, timed ‘up and go’, muscle strength, and range of motion between both groups

		Immediately after intervention			After 2 days		
Variables	Group	Change from baseline mean (95% CI)	Difference in mean change (95% CI)	*p*-value	Change from baseline mean (95% CI)	Difference in mean change (95% CI)	*p*-value
VAS (cm)	MWM Sham	−2.7 (−3.1, − 2.2) − 0.5 (− 0.9, − 0.0)	− 2.2 (− 2.8, − 1.6)	< 0.001^*^	− 0.9 (− 1.5, − 0.3) 0.1 (− 0.5, 0.7)	−1.0 (− 1.8, − 0.1)	0.026^*^
PPT knee (kPa)	MWM Sham	185 (131, 240) 10 (− 45, 64)	176 (97, 254)	< 0.001^*^	65 (29, 102) 27 (− 9, 63)	39 (− 14, 91)	0.142
PPT shoulder (kPa)	MWM Sham	209 (155, 263) − 3 (−57, 51)	212 (136, 288)	< 0.001^*^	106 (58, 154) − 2 (− 49, 46)	107 (40, 175)	0.003^*^
TUG (seconds)	MWM Sham	−1.6 (2.0, − 1.2) 0.0 (− 0.4, 0.4)	−1.6 (− 2.1, − 1.1)	< 0.001^*^	− 0.9 (− 1.3, − 0.5) − 0.0 (− 0.4, 0.4)	−0.9 (− 1.4, − 0.4)	0.001^*^
HHD knee flexion (kg)	MWM Sham	2.5 (2.0, 3.0) 0.5 (− 0.1, 1.0)	2 (1.3, 2.7)	< 0.001^*^	1.1 (0.6, 1.6) 0.2 (− 0.4, 0.7)	0.9 (0.2, 1.7)	0.018^*^
HHD knee extension (kg)	MWM Sham	6.0 (5.0, 7.1) 0.4 (−0.7, 1.5)	5.7 (4.1, 7.2)	< 0.001^*^	3.3 (2.7, 4.0) 0.3 (−0.3, 1.0)	2.9 (2.1, 3.9)	< 0.001^*^
ROM knee flexion (°)	MWM Sham	15.1 (12.9, 17.4) 2.4 (0.2, 4.6)	12.8 (9.6, 15.9)	< 0.001^*^	10.2 (7.7, 12.7) 1.9 (− 0.6, 4.4)	8.3 (4.7, 11.9)	< 0.001^*^
ROM knee extension (°)	MWM Sham	−0.6 (−1.2, − 0.1) 0.1 (− 0.5, 0.7)	−0.8 (− 1.6, 0.1)	0.067	−0.3 (− 0.9, 0.3) − 0.0 (− 0.6, 0.5)	−0.3 (− 1.1, 0.5)	0.499

*CI* Confidence interval, *HHD* Hand-held dynamometer, *MWM* Mobilization with movement, *ROM* Range of motion, *PPT* Pressure pain threshold, *TUG* Timed “Up and Go”, VAS Visual analogue scale

^*^Significance difference (*p* < 0.05)

**Table 4:** Comparison of the Western Ontario and McMaster Universities Osteoarthritis Index between both groups

Variables	Group	Change from baseline mean (95% CI)	Difference in mean change (95% CI)	*p*-value
Pain scale	MWM Sham	- 0.2 (− 1.1, 0.9) - 0.1 (− 0.9, 0.7)	- 0.1 (− 1.3, 1.0)	0.813
Stiffness scale	MWM Sham	0.0 (− 0.4, 0.5) - 0.1 (− 0.5, 0.3)	0.1 (− 0.5, 0.7)	0.700
Function scale	MWM Sham	0.3 (− 1.9, 2.4) 1.6 (− 0.6, 3.7)	- 1.3 (− 4.4, 1.8)	0.392
Total score	MWM Sham	- 0.2 (− 3.1, 2.7) 1.6 (− 1.3, 4.4)	- 1.8 (− 5.9, 2.4)	0.396

*CI* Confidence interval, *MWM* Mobilization with movement

## Discussion

This study investigated the immediate and short-term effects of MWM compared to sham MWM on function and local and widespread pain in patients with knee OA. MWM resulted in an immediate reduction in pain as measured by VAS. The mean difference in VAS scores was 2.7 cm and 0.9 cm immediately post-intervention and after 2 days, respectively, more than the ‘minimal clinically relevant’ difference of 0.84 cm [48]. This effect was similar to previously recorded results for ankle sprains [49], de Quervain’s tenosynovitis [50], lateral epicondylalgia [51] and hip OA [21]. This reduction of pain lasted for 2 days. A similar result was found in patients with knee OA where MWM was applied in a case series [23] or in RCT’s where MWM was used in combination with other treatments [24, 25].

A reduction of mechanical pain, as measured by an algometer, was also observed following MWM, as demonstrated by increased PPTs in the knee. This result is similar to the findings of studies of spinal mobilization [31, 52] and peripheral joint mobilization of the elbow [53] and knee OA [22]. Interestingly, in this study, an improvement in PPT was seen in the distant area (i.e. shoulder) in the treatment group but not in the sham group. The increase in PPT was > 15% immediately post-intervention (for knee and shoulder) and after 2 days (for the shoulder), which is considered to reflect a clinically significant effect [22]. Previous studies revealed that mobilization of the cervical spine decreases hyperalgesia in the upper limbs [31, 54] and that knee mobilization induces hypoalgesic responses down to the heel [22].

Research has shown that joint mobilization not only initiates local physiological mechanisms but also involves central mechanisms such as facilitation of inhibitory pathways in the spinal cord or descending inhibitory pathways from higher levels in the brainstem [22]. Skyba et al. [55] reported that serotonergic and noradrenergic receptors in the spinal cord mediate analgesia produced by knee joint mobilization.

Knee flexion ROM improved significantly immediately after intervention with MWM in this study. This result corresponds to previous studies of the knee and hip. A case series [23] and RCT [24] reported improvement of knee flexion ROM following MWM in patients with knee OA. Beselga et al. [21] reported immediate improvement of hip flexion and internal rotation ROM following a single treatment of MWM in patients with hip OA.

The present study demonstrated an immediate and short-term effect of knee MWM on motor activity, as indicated by significant improvements in knee flexor and extensor muscle strength. These improvements may be due to the reversal of reflex pain inhibition [56]. Alteration in motor activity may also be an indication of a response that is mediated at the level of the central nervous system [56]. MWM improved quadriceps muscle strength significantly in patients with knee OA up to 1-year follow-up [24]. Mobilization of the cervical spine improved the function of deep neck flexor in patients with neck pain [52] and increased pain-free grip strength in patients with lateral epicondylalgia [16, 31].

In this study, MWM improved TUG time. The decrease in time needed to walk 6 m was 1.6 s immediately after the intervention, which is considered to reflect a clinically significant effect [42]. Our finding is consistent with the study by Altmış et al. [25]. In patients with hip OA, Beselga et al. [21] found that MWM reduced the time needed to walk 6 m in this functional test. However, another manual therapy technique, namely antero-posterior glide, had no effect on this test in patients with knee OA [22]. This disagreement may be attributed to the different mobilization techniques used, test procedures and/or the characteristics of the patients in the two studies. These contradictory findings emphasize the need of further research in this area. In this study, several patients received MWM in weight-bearing positions. Thus, patients simultaneously received self-feedback from their painless joint movement.

While the reduction of pain and the improvement of physical function were achieved by MWM, the WOMAC Index scores did not change. This may be because the grade of OA (on the K&L scale) was relatively low, which may represent a non-major limitation of functional activity. Moreover, 2 days might not be sufficient for a perceived improvement in daily activities. Moss et al. [22] reported no improvement in WOMAC Index scores after the initial effect of antero-posterior glide in patients with knee OA. However, longer sessions of MWM or other manual therapy techniques in combination with exercise produced significant improvements in WOMAC Index scores in other studies [24, 57, 58].

A strength of this study is that a sham treatment was used, which is considered more appropriate than no or usual treatment as a control. A limitation of is the short-term design, which may suggest that the immediate changes of any outcome cannot be extrapolated to long-term changes. However, significant improvements in pain, function, ROM and muscle strength were noted in this study, as in previous studies [21–23].

## Conclusion

The current study suggests that MWM but not sham MWM for patients with knee OA provides a local and widespread hypoalgesic effect, increases knee flexion ROM, increases knee flexor and extensor strength and improves physical function. Although this study demonstrated immediate and short-term effects that persisted for 2 days after the intervention, more research is needed to determine the long-term efficacy of this approach.

## Data Availability

The datasets used and/or analyzed during the current study are available from the corresponding authors on reasonable request.

## Abbreviations

ANCOVA: Analysis of covariance
CI: Confidence interval
ES: Effect size
ICCs: Intraclass correlation coefficients
IRB: Institutional review board
K&L: Kellgren and Lawrence
KFHU: King Fahd Hospital of the University
LMM: Linear mixed model
MWM: Mobilization with movement
OA: Osteoarthritis
PPT: Pressure pain threshold
RCTs: Randomized controlled trials
ROM: Range of motion
TUG: Timed “Up and Go”
VAS: Visual analogue scale
WOMAC: Western Ontario and McMaster Universities
